# An Improved Seeker Optimization Algorithm for Phase Sensitivity Enhancement of a Franckeite- and WS_2_-Based SPR Biosensor for Waterborne Bacteria Detection

**DOI:** 10.3390/mi15030362

**Published:** 2024-03-03

**Authors:** Chong Yue, Xiuting Zhao, Lei Tao, Chuntao Zheng, Yueqing Ding, Yongcai Guo

**Affiliations:** 1 College of Optoelectronic Engineering, Chongqing University, Chongqing 400030, China; ycguo@cqu.edu.cn; 2 Chongqing Academy of Metrology and Quality Inspection, Chongqing 401123, China; taol_cqjz@163.com (L.T.); yueqing_ding@163.com (Y.D.); 3 College of Advanced Manufacturing, Guangdong University of Technology, Jieyang 510008, China; chuntao.zheng@gdut.edu.cn

**Keywords:** SPR, waterborne bacteria, phase sensitivity, improved seeker optimization algorithm, Franckeite, WS_2_

## Abstract

For the purpose of detecting waterborne bacteria, a high-phase-sensitivity SPR sensor with an Ag–TiO_2_–Franckeite–WS_2_ hybrid structure is designed using an improved seeker optimization algorithm (ISOA). By optimizing each layer of sensor construction simultaneously, the ISOA guarantees a minimum reflectance of less than 0.01 by Ag (20.36 nm)–TiO_2_ (6.08 nm)–Franckeite (monolayer)–WS_2_ (bilayer) after 30 iterations for *E. coli*. And the optimal phase sensitivity is 2.378 × 10^6^ deg/RIU. Sensor performance and computing efficiency have been greatly enhanced using the ISOA in comparison to the traditional layer-by-layer technique and the SOA method. This will enable sensors to detect a wider range of bacteria with more efficacy. As a result, the ISOA-based design idea could provide SPR biosensors with new applications in environmental monitoring.

## 1. Introduction

Surface plasmon resonance (SPR) biosensors utilize plasmon waves to measure alterations in the refractive index occurring on the sensing surface [1]. Numerous benefits of the SPR biosensor include its low cost, straightforward design, excellent sensitivity, label-free detection, and real-time characteristics [2,3,4]. Its potential applications are extensive and include biological detection, food safety, environmental pollution detection, and other domains [5,6,7]. Therefore, several researchers have carried out in-depth investigations using novel modified configurations [8,9] and modifying the structure [10] to promote SPR biosensor’s performance [11,12].

Because phase sensitivity configuration is less sensitive to external impacts, it may be utilized to improve the sensitivity of SPR biosensors [13,14,15]. The fact that the phase of the incident light wave’s transverse magnetic (TM) polarized component varies significantly while the phase of the transverse electric (TE) stays mostly constant serves as the foundation for this configuration [16,17]. Silver (Ag) is favored as an active metal for SPR sensors due to its lower D-electron energy bands and bulk plasma frequency [18,19,20]. However, the oxidation susceptibility of the silver film at room temperature affects the sensor’s performance. This issue is resolved by using a guided wave structure, which is a thin dielectric nanolayer with a high dielectric constant on the metal film, to boost the SPR biosensor’s sensitivity [21,22,23,24]. For example, Deng used titanium dioxide (TiO_2_) film in a SPR gas sensor and obtained high sensitivity in hydrogen detection at the telecommunications wavelength [25]. However, the waveguide layer also widens the dip of reflectivity and decrease its depth [26,27]. So to overcome this obstacle, two-dimensional (2D) materials have been employed to increase light absorption and compatibility with biological systems. 

Franckeite belongs to the sulfosalt family, and is a van der Waals heterostructure stabilized in its natural state. It is made up of stacks of alternating PbS pseudotetragonal and SnS_2_-like pseudohexagonal layers [28,29]. In addition, Franckeite is characterized by a narrow bandgap of less than 0.7 eV, a rare feature in 2D materials, with p-type semiconductor properties [30]. It has a crystalline structure and is reported to be stable in air. The combination of these features is rare, resembling those seen in just a few of 2D materials, such as black phosphorus and tungsten diselenide. Unlike black phosphorus, Franckeite exhibits stability in the surrounding environment [31]. Due to the aforementioned characteristics, Franckeite shows significant promise for application in optoelectronic devices. Gan et al. has proposed a SPR biosensor with a Ag–Franckeite–graphene hybrid structure, and obtained the highest sensitivity as 188 deg/RIU [32].

Furthermore, there has been significant interest in transition metal dichalcogenides like molybdenum disulfide (MoS_2_) and tungsten disulfide (WS_2_) due to their exceptional electron mobility, better surface volume ratio, and high dielectric constant [33,34,35]. Zeng has presented a SPR biosensor with a phase sensitivity of 8.185 × 10^4^ deg/RIU based on the graphene-MoS_2_ structure [36]. Likewise, Han proposed an SPR sensor with a Ag–ITO–WS_2_ configuration, which achieved a maximum phase sensitivity of 1.711 × 10^6^ deg/RIU [37]. Despite the progress made in sensitivity improvement, the traditional layer-by-layer optimization approach employed in these studies has been deemed inefficient for addressing multi-objective and multi-variable optimization issues when the number of SPR sensor layers rises. Consequently, the conventional methods based on direct assessment of targets under different values of variables become less effective. Hence, it is imperative to concurrently adjust the thickness of all layers in the biosensor to address these difficulties.

Hence, intelligent optimization algorithms have been created to produce a multi-layer SPR biosensor with enhanced sensitivity and resolution [38]. Amoosoltani proposed the particle swarm optimization (PSO) algorithm to optimize the thickness of metal thin films in SPR sensors, and the results show that the PSO algorithm has certain advantages in obtaining high-performance sensors by optimizing copper film thickness [39]. Further, Sun applied the PSO algorithm to SPR biosensors in four different modulation modes (wavelength, angle, intensity, and phase) [40], and the findings demonstrate that the PSO-based optimization structure outperforms the experimental structure. In addition, Lin optimized the sensor’s thickness, resulting in enhanced angular sensitivity within the visible spectrum [41]. However, there is still room for improvement of the SPR sensor based on phase modulation, as performance depends not only on the phase sensitivity, but also on the minimum reflectivity at resonance. The seeker optimization algorithm (SOA) is a swarm intelligence algorithm proposed by Dai and Chen in 2006 [42]. The SOA directly uses a range of good human social behaviors for modelling and analysis, such as individuals evolving into good individuals, good individuals evolving into good groups, and good groups evolving into good populations. All individuals participate in the search, determining the direction and step size of the search through individuals to update their position and obtain the optimal solution within their range [43]. However, in the SOA, the historical optimal fitness of all searchers in the population is calculated, and then ranked from high to low to form a linear affiliation function, which increases the complexity of the optimization computation. In addition, in the basic SOA, there is a need for later search steps as long processing is not precise enough. In addition, the basic SOA does not have measures to break away from local optima, which can easily lead to premature maturation [44]. In response to the above issues, an improved seeker optimization algorithm (ISOA) is introduced to simultaneously change all of these parameters. In contrast to the SOA, its adaptive search step effectively avoids bypassing the valley region, rendering it well suited for parallel computing and capable of handling a substantial number of design parameters.

This article utilizes the ISOA approach to create SPR sensors using a Ag–TiO_2_–Franckeite–WS_2_ structure. The sensor is specifically engineered to possess a heightened phase sensitivity to detect bacteria in water. The ISOA approach utilizes an objective function that ensures a minimum reflectance of less than 0.01. By tweaking the thickness of each layer in the Ag–TiO_2_–Franckeite–WS_2_ structure at the same time, we can enhance the efficacy of the sensor in detecting waterborne bacteria and also reduce the time required for designing.

The rest of this paper is organized as follows. First of all, the theory and design methodology of sensor structure is described in Section 2. Subsequently, the principle and improvement strategy of the SOA are discussed in Section 3. Then, in Section 4, the result of the simulation is presented. At last, the conclusion is presented in Section 5. 

## 2. Theory and Design Methodology

**Figure 1:**
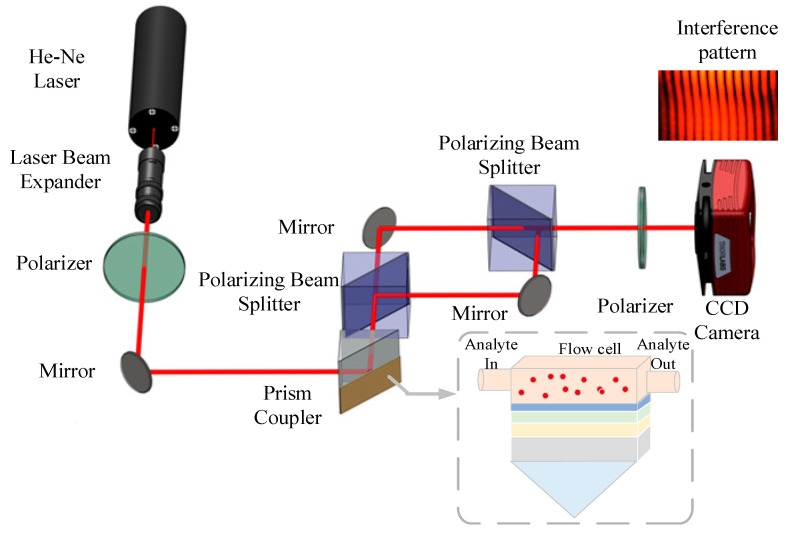
The phase-sensitive SPR setup schematic for the detection of bacteria in water.

The schematic design of a phase-sensitive SPR system for water bacterium identification is depicted in Figure 1. The proposed biosensor consists of six layers: BK7 ($n_{BK7}=1.5151$), Ag ($n_{Ag}=0.0803+4.2347i$), TiO_2_ ($n_{TiO_{2}}=2.5837$), Franckeite, WS_2_ and sensing medium. The data provided in Table 1 summarize the characteristic parameter of 2D materials at 633 nm. The sensing medium comprises three categories of waterborne bacteria: pure water, *V. cholera*, and *E. coli*. The refractive indices (RIs) for each type are presented in Table 2. The viability of the suggested approach in the experiment is demonstrated in Figure 1, which forms the basis for the numerical simulation technique. To attain a linear polarization angle of 45° for both TM and TE waves, the He-Ne laser undergoes polarization by passing through a polarizer. Subsequently, the SPR biosensor is exposed to light within a defined range of angles. To accurately assess the phase sensitivity of the SPR biosensor, input the interference structure to determine the exact phase difference.

To calculate the reflectance of reflected light of a multi-layer structural model, the transfer matrix approach is utilized. The characteristic matrix is described as follows [48]:

(1) $$M=\prod_{m=1}^{N}M_{m}=\left[\begin{array}{cc} \cos\beta_{m} & -\frac{i}{q_{m}}\sin\beta_{m}\\ -q_{m}\sin\beta_{m} & \cos\beta_{m} \end{array}\right]$$

with

(2) $$\left\{\begin{array}{ll} q_{k}=\frac{{\left(\varepsilon_{k}-n_{0}^{2}\sin^{2}\theta_{0}\right)}^{\frac{1}{2}}}{\varepsilon_{k}} & TM-wave\\ q_{k}={\left(\varepsilon_{k}-n_{0}^{2}\sin^{2}\theta_{0}\right)}^{\frac{1}{2}} & TE-wave \end{array}\right.$$

(3) $$\beta_{m}=\frac{2\pi d_{m}}{\lambda }{\left(\varepsilon_{m}-n_{0}^{2}\sin^{2}\theta_{0}\right)}^{\frac{1}{2}}$$

where $\beta_{m}$ and $q_{k}$ represent the phase factor and optical admittances, respectively. $\varepsilon_{m}$ and $\theta_{0}$ are the dielectric permittivity and the angle of incidence.

The reflection coefficient, denoted as *r*, can be determined for both TM waves and TE waves using the following formula:

(4) $$r=\frac{\left(M_{11}+M_{12}q_{N})q_{0}-(M_{21}+M_{22}q_{N}\right)}{\left(M_{11}+M_{12}q_{N})q_{0}+(M_{21}+M_{22}q_{N}\right)}$$

Therefore, the reflectivity of TM waves and the phase difference between TM waves and TE waves are obtained as:

(5) $$R_{TM}={\left|r_{TM}\right|}^{2}$$

(6) $$\phi_{d}=\left|\phi_{TM}-\phi_{TE}\right|$$

The biosensor’s phase sensitivity is described as follows:

(7) $$S=\frac{\Delta \phi_{d}}{\Delta n_{bio}}$$

## 3. The Improved Seeker Optimization Algorithm

The SOA is a kind of heuristic stochastic search algorithm proposed in recent years, The SOA directly analyses the stochastic search behaviors of humans, and analyses and researches the behaviors of humans as high-level agents, mainly with the help of the latest research results of brain science, agent systems, artificial intelligence and cognitive science in human research [49]. Unlike existing optimization algorithms, the SOA simulates human intelligent search behavior, where each individual is considered the optimal individual, but individuals have good communication, collaboration, learning, and reasoning abilities, search teams are used as the population and the seeker’s position is used as the candidate solution in the SOA, which mimics human intelligent search behavior. By simulating the human search for “experience gradients” and uncertain reasoning, the optimal solution to the problem is attained. Nevertheless, the SOA has limitations, such as low search accuracy and a tendency to become locked in the local optimum. An ISOA based on the adaptive membership degree is created to address these issues. Since it avoids skipping valley areas by decreasing search step sizes in the middle and late stages of the algorithm, it is suitable for multi-objective optimization.

The following are the procedures involved in creating a phase-sensitive SPR biosensor using the ISOA for waterborne bacteria detection. The population locations are first established randomly. Subsequently, adaptive search step and search direction operations are carried out on the updated positions of each seeker [50]:

(8) $$\alpha_{ij}=\omega abs(x_{\min}-x_{\max})\sqrt{-\ln(u_{ij})}$$

where $\alpha_{ij}$ represents the search step, $\mu_{ij}$ is the degree of membership, the inertia weight is denoted by ω, and $x_{\min}$ and $x_{\max}$ correspond to the minimum and maximum objective function values.

(9) $$u_{ij}=\mathrm{rand}(u_{i},1),\;\;j=1,\ldots D$$

(10) $$u_{i}=u_{\min}+(u_{\max}-u_{\min})*{\left(\frac{t}{T}\right)}^{\left(1-\frac{t}{T}\right)}$$

In the ISOA, the degree of membership is modified solely by the number of iterations *t*. Additionally, an adaptive power is introduced based on the linear degree of membership, as depicted in Equations (9) and (10), where *T* represents the maximum number of iterations. This adaptation causes the rate of change in the degree of membership to decrease with increases in iterations. Consequently, the algorithm undergoes a relatively small search step during the middle and late phases, effectively addressing the issue of the algorithm being susceptible to becoming stuck in the local optimum.

Moreover, search direction can be expressed as:

(11) $$d_{ij}(t)=sign(\omega d_{i,pro}+\varphi_{1}d_{i,ego}+\varphi_{2}d_{i,alt})$$

where $\varphi_{1}$ and $\varphi_{2}$ are random real numbers on the interval of [0, 1]; $d_{i,ego}$, $d_{i,alt}$ and $d_{i,pro}$ represent self-interest direction, altruism direction and pre-action direction, respectively.

Ultimately, by using the aforementioned procedure to update the position of the seeker, new populations are created. Subsequently, these populations are then assessed, and until the termination condition is met, the global optimum is amended again. Algorithm 1 provides a detailed description of the ISOA’s pseudocode.
**Algorithm 1: ISOA** Initialization: (1) Population N, dimension D, generation T, inertia weight ω, degree of membership *u*_min_, *u*_max_ (2) Randomly initialize seeker position x, $\varphi_{1}$ and $\varphi_{2}$, *P*_i_ and *P*_g_ of seeker (3) Cycle (4) **For** i = 1:N (5)  **For** j = 1:D (6)   $d_{ij}(t)=sign(\omega d_{i,pro}+\varphi_{1}d_{i,ego}+\varphi_{2}d_{i,alt})$ (7)   $u_{i}=u_{\min}+(u_{\max}-u_{\min})*{\left(\frac{t}{T}\right)}^{\left(1-\frac{t}{T}\right)}$ (8)   $u_{ij}=\mathrm{rand}(u_{i},1)$ (9)   $\alpha_{ij}=\omega abs(x_{\min}-x_{\max})\sqrt{-\ln(u_{ij})}$   % Update the position of seeker (10)   $\Delta x_{i,j}(t+1)=\alpha_{ij}(t)d_{ij}(t)$ (11)   $x_{ij}(t+1)=x_{ij}(t)+\Delta x_{ij}(t+1)$   % Update pbest and gbest value (12)    **IF** func($x_{ij}$) > func($p_{ij}$) **then**
$p_{ij}=x_{ij}$ (13)    **End IF** (14)    **IF** func($x_{ij}$) > func($p_{gj}$) **then**
$p_{gj}=x_{ij}$ (15)    **End IF**
(16)  **End** (17) **End**

The ISOA employs an objective function (*OF*) with a constrained function to achieve multi-objective optimization and obtain high phase sensitivity and low reflectivity:

(12) $$OF=\left\{\begin{array}{l} S\;,R_{\min}<0.01\\ 0,\;others \end{array}\right.$$

The objective function is designed to a identify maximum value of *S* within the search area, with the objective of indirectly minimizing the value of *R*_min_, which represents the minimum reflectivity at the resonance angle. *R*_min_ above 0.01 will result in discarding the solution.

## 4. Results and Discussion

**Figure 2:**
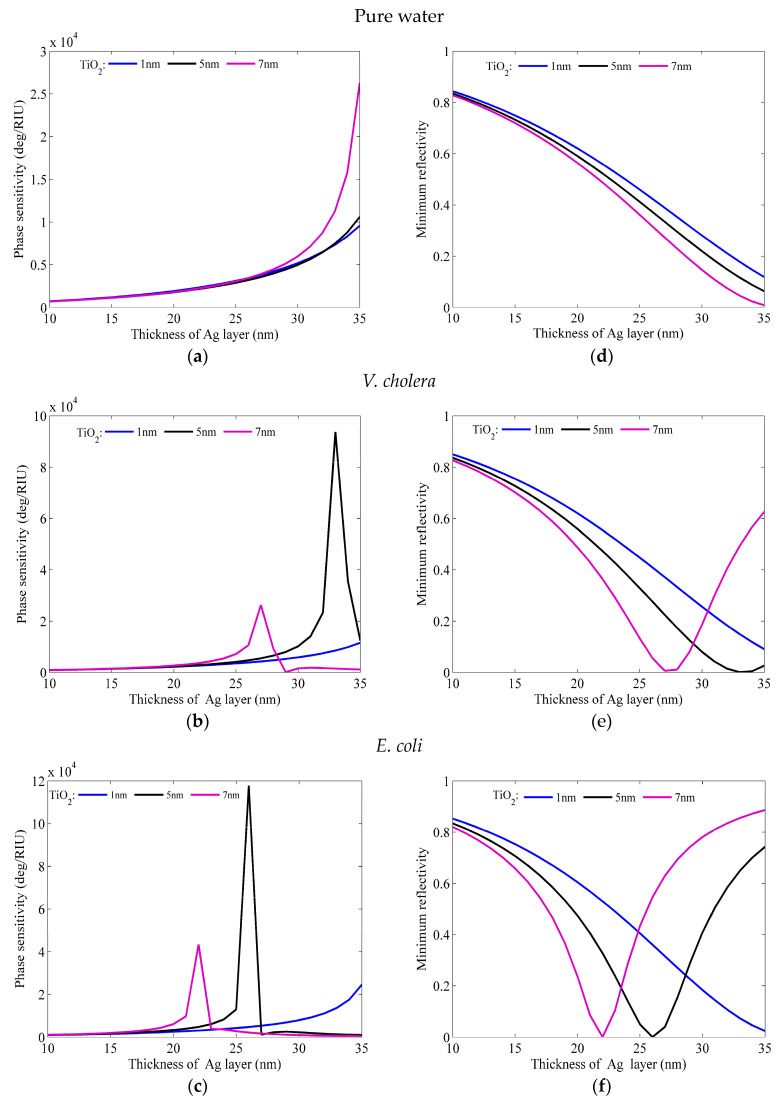
The phase sensitivity as a function of the thickness of the Ag layer and TiO_2_ (1 nm, 5 nm, 7 nm) for different sensing mediums: (**a**) pure water, (**b**) *V. cholera*, and (**c**) *E. coli*; and corresponding change in minimum reflectivity with the various thicknesses of Ag and TiO_2_ (1 nm, 5 nm, 7 nm) in (**d**) pure water, (**e**) *V. cholera*, and (**f**) *E. coli*.

To validate the effectiveness of the methods presented in this paper, the conventional method and the SOA method are used to optimize and validate the same sensor structures. In the conventional layer-by-layer optimization approach, the primary objective is to optimize the thickness of Ag and TiO_2_ at the monolayer of Franckeite and WS_2_. To achieve the best possible thickness combination of Ag and TiO_2_ film, it is important to minimize the *R*_min_ and maximize the phase sensitivity. Thus, when the sensing medium is waterborne bacteria, Figure 2 illustrates the change in phase sensitivity and minimum reflectivity with the different thicknesses of Ag (10 nm~35 nm) and TiO_2_ (1 nm, 5 nm, 7 nm). As seen in Figure 2, the phase sensitivity increases monotonically from 10 nm to 35 nm. At 35 nm for Ag thickness and 7 nm for TiO_2_ thickness, the maximum phase sensitivity of pure water is 2.631 × 10^4^ deg/RIU, and the lowest reflectance is 8.783 × 10^−3^. The maximum phase sensitivity of *V. cholera* is 9.374 × 10^4^ deg/RIU, and the Rmin is 9.739 × 10^−4^ at 33 nm and 5 nm thicknesses of Ag and TiO_2_. The ideal Ag and TiO_2_ layer thicknesses for *E. coli* are 26 nm and 5 nm, respectively. The lowest reflectivity is 4.893 × 10^−4^ and the maximum sensitivity is 1.178 × 10^5^ deg/RIU.

**Figure 3:**
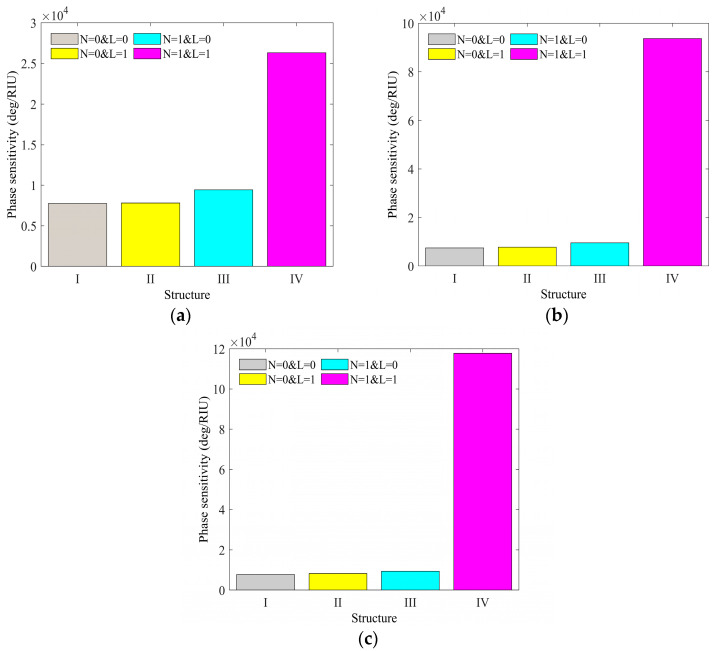
The phase sensitivity in accordance with other sensor structures at the optimum thickness of Ag and TiO_2_ in (**a**) pure water, (**b**) *V. cholera*, and (**c**) *E. coli*.

Furthermore, to provide additional evidence of the enhanced phase sensitivity attained by the amalgamation of the Franckeite and WS_2_ layer, Figure 3 illustrates the phase sensitivity of another sensor construction at the optimal thickness of Ag and TiO_2_. From the figure, it is evident that the sensor without the Franckeite layer and WS_2_ layer (N = 0 and L = 0) exhibits the lowest phase sensitivity. The addition of either a monolayer of Franckeite (N = 1 and L = 0) or WS_2_ layer (N = 0 and L = 1) does not considerably enhance the phase sensitivity of the sensor. The performance of sensors could be significantly enhanced by including both a monolayer of Franckeite and WS_2_. This suggests that the sensor structure presented in this paper is more efficient in detecting water bacteria compared to conventional architectures.

**Figure 4:**
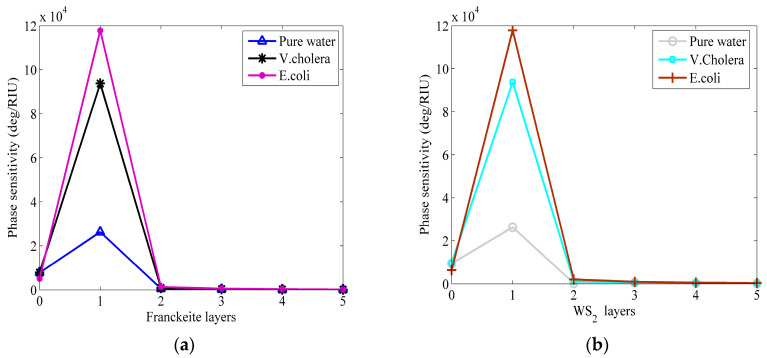
Phase sensitivity varies in relation to different layers of (**a**) Franckeite and (**b**) WS_2_.

Moreover, Figure 4 illustrates the relationship between phase sensitivity and the number of Franckeite layers in the WS_2_ monolayer, as well as the number of layers in the Franckeite monolayer. One can visualize from the figure that both the Franckeite and WS_2_ layers are monolayers, and the sensor can obtain the best phase sensitivity value for the detection of waterborne bacteria at the optimum thickness of Ag and TiO_2_.

Based on the analysis provided, it can be inferred that the sensor structure incorporating Franckeite and WS_2_ layers is viable for enhancing phase sensitivity. However, the conventional approach of optimizing the layers one by one is computationally demanding and ineffective. Hence, it is crucial to concurrently tune every layer thickness of the sensor utilizing algorithms.

An Ag–TiO_2_–Franckeite–WS_2_ structure-based SPR biosensor for detecting the water bacteria was designed via the SOA and the ISOA to validate the feasibility of the method. From the theoretical model, we can see that the OF is determined by the thickness of Ag (*d*_1_) and TiO_2_ (*d*_2_), the layer of Franckeite (*N*) and WS_2_ (*L*), where $d_{1}\in [0,50]$, $d_{2}\in [0,50]$, N∈(0,10], and L∈(0,10]. The search range is based on experience in order to obtain good sensing performance. Initialization parameter settings are provided in Table 3 prior to execution of the algorithm.

**Figure 5:**
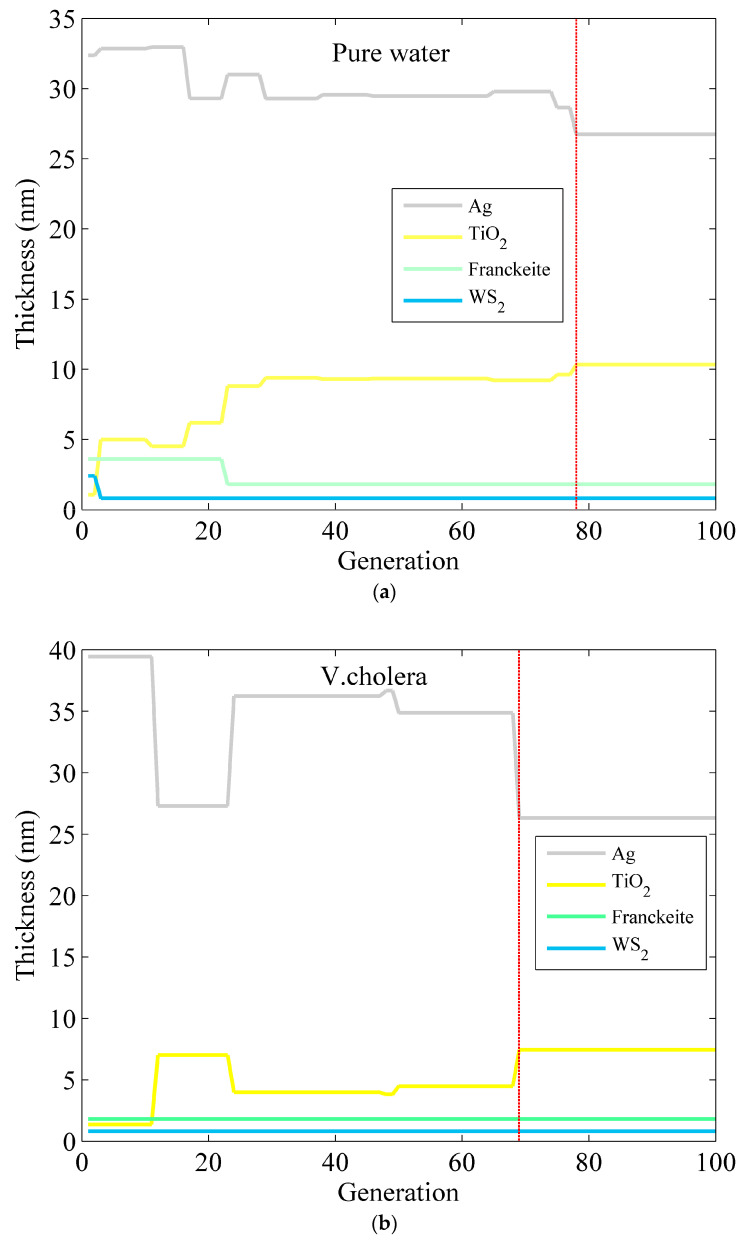

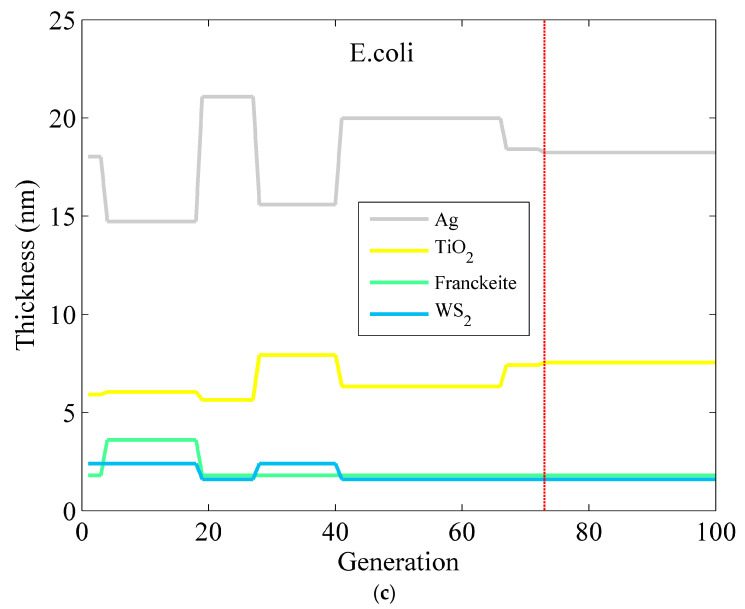
The optimized thickness of the Ag–TiO_2_–Franckeite–WS_2_ structure via the SOA for detection in (**a**) pure water, (**b**) *V. cholera*, and (**c**) *E. coli*.

After 100 iterations, the SOA method is employed to optimize the layer thickness of the Ag–TiO_2_–Franckeite–WS_2_ structure for the detection of waterborne bacteria, as seen in Figure 5, the red line represents the location of the optimal parameters mentioned in Table 4. Simultaneously, Table 4 displays the relevant characteristics such as the minimum reflectivity, maximum phase sensitivity, and number of iterations. After 79 iterations, the optimal phase sensitivity for detecting pure water is obtained when the thickness of the Ag and TiO_2_ layers is 26.75 nm and 10.33 nm, respectively. The Franckeite and WS_2_ layers are both monolayers. The greatest phase sensitivity reached is 1.841 × 10^6^ deg/RIU, while the lowest reflectivity value is 3.373 × 10^−6^. For *V. cholera*, the maximum phase sensitivity and minimum reflectivity are 1.909 × 10^6^ deg/RIU and 2.307 × 10^−7^ after 70 iterations, when the Ag is 26.30 nm, TiO_2_ is 7.45 nm, and Franckeite and WS_2_ are monolayers. At last, the optimization of the Ag–TiO_2_–Franckeite–WS_2_ structure for *E. coli* detection is achieved by using a one-layer Franckeite, a bilayer WS_2_, a 18.23 nm-thick Ag layer, and a 7.54 nm-thick TiO_2_ layer. The greatest phase sensitivity value obtained is 2.355 × 10^6^ deg/RIU. Simultaneously, the minimum reflectivity is 9.455 × 10^−6^. Based on the aforementioned findings, it is evident that the phase sensitivity of the sensing structure may be significantly enhanced, by a factor of 1~2 orders of magnitude, compared to the usual technique. This improvement is achieved by optimizing the thickness of each sensor layer using an algorithm.

**Figure 6:**
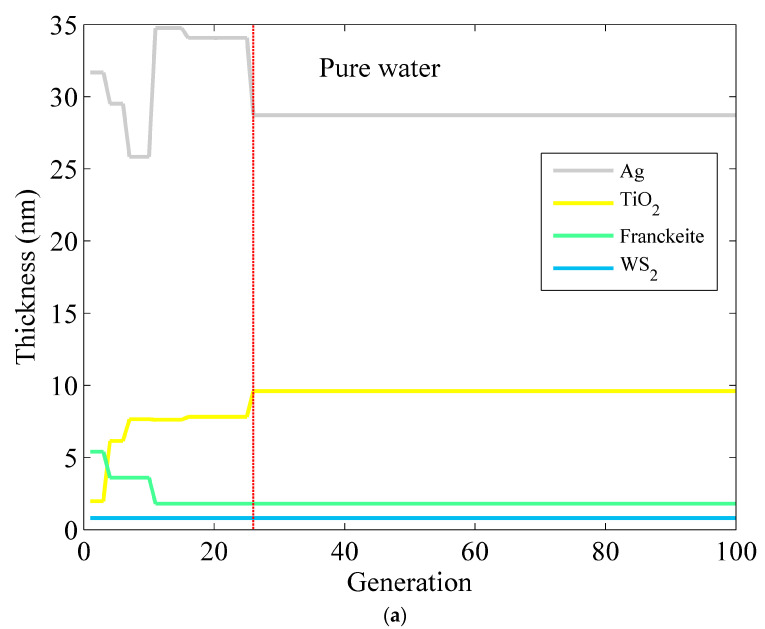

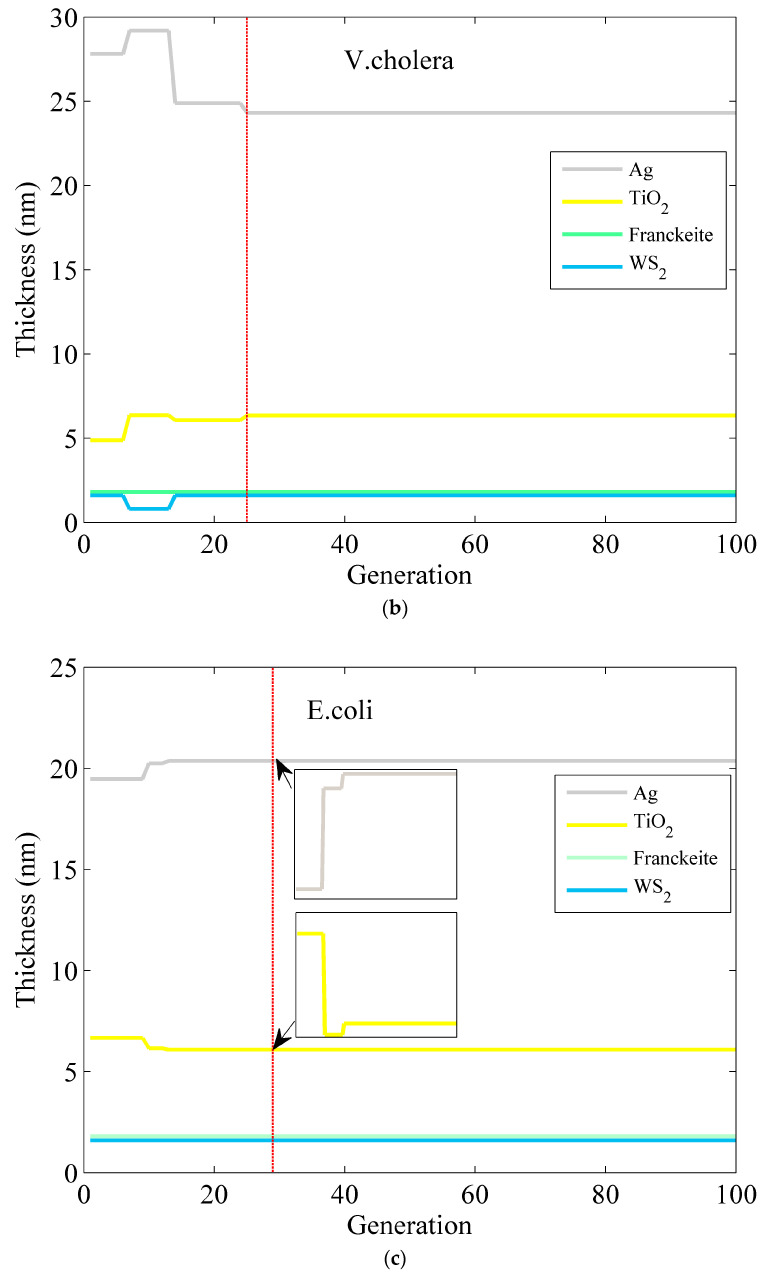
The optimized thickness of the Ag–TiO_2_–Franckeite–WS_2_ structure via the ISOA for detection in (**a**) pure water, (**b**) *V. cholera*, and (**c**) *E. coli*.

Meanwhile, Figure 6 and Table 5 give the optimum layer thickness, the red line represents the location of the optimal parameters mentioned in Table 5, phase sensitivity and reflectivity of Ag–TiO_2_–Franckeite–WS_2_ for detecting the waterborne bacteria by the ISOA. With a guaranteed minimum reflectance of less than 0.01, it is evident that phase sensitivity based on the ISOA is greatly improved compared to base on the SOA and traditional techniques. Furthermore, the required number of algorithm iterations to obtain a stable optimal value is significantly decreased. The phase sensitivity of pure water may attain a maximum value of 1.871 × 10^6^ deg/RIU. The minimum reflectance occurs at a value of 2.058 × 10^−6^ when the Ag layer has a thickness of 28.72 nm, the TiO_2_ layer has a thickness of 9.59 nm, and there are 27 iterations, with one layer each of Franckeite and WS_2_. Subsequently, optimized Ag–TiO_2_–Franckeite–WS_2_ for detecting the *V. cholera* is obtained after 23 iterations. The phase sensitivity reaches a maximum value of 1.950 × 10^6^ deg/RIU when the thickness of Ag is 24.31 nm, that of TiO_2_ is 6.34 nm, the Franckeite is in a monolayer configuration, and the WS_2_ is in a bilayer configuration. Finally, the greatest phase sensitivity is 2.378 × 10^6^ deg/RIU, while the lowest reflectivity is 1.307 × 10^−6^. The presence of a single layer of Franckeite and a bilayer of WS_2_, with a Ag thickness of 20.36 nm and a TiO_2_ thickness of 6.08 nm, enables the detection of *E. coli*. By comparing the optimization data of the SOA and the ISOA, it is clear that the ISOA method exhibits improved convergence qualities since it needs fewer iterations to find the best solution. Furthermore, the ISOA method has an improved global search capability that allows it to bypass the local optimum and take full advantage of the high electron mobility of Franckeite and WS_2_. This improves the ability of SPR sensors to detect small changes in phase and makes them suitable for detecting bacteria present in water. The ISOA technique has the advantage of efficiently managing many significant design parameters by simultaneously determining the optimal thickness of each layer. This results in time savings, particularly when dealing with a larger number of SPR sensor layers.

**Figure 7:**
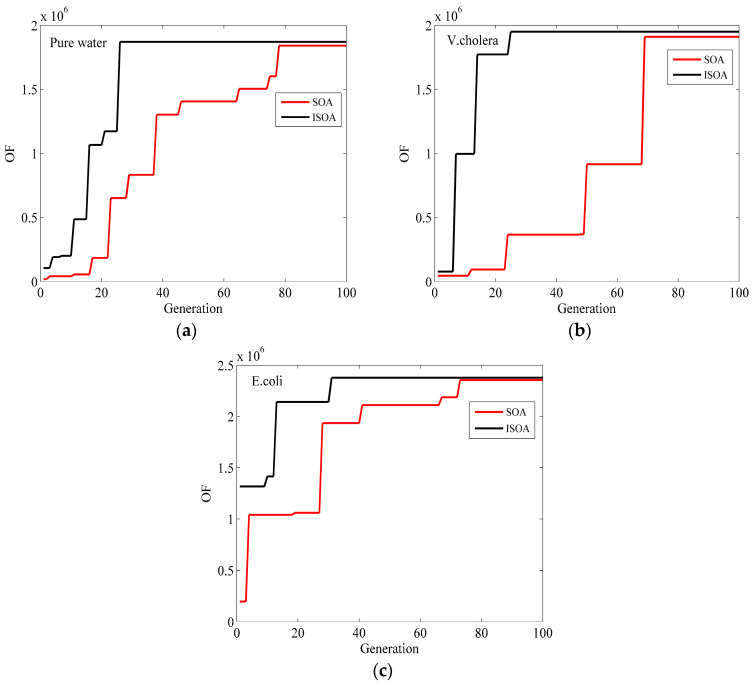
The optimized OF change in the Ag–TiO_2_–Franckeite–WS_2_ structure varies across the generation of the ISOA and the SOA in (**a**) pure water, (**b**) *V. cholera*, and (**c**) *E. coli*.

Figure 7 illustrates the curves of the objective function for the Ag–TiO_2_–Franckeite–WS_2_ structure of the SPR biosensor. These curves are used to identify waterborne bacteria and are obtained by employing both the SOA and the ISOA. The curve indicates that the merit function increases at a faster rate during iterations with the ISOA as opposed to the standard SOA. Furthermore, for the ISOA, the merit function stabilizes at a higher level after approximately 30 iterations, while the standard SOA takes approximately 80 iterations to achieve the same stabilization. This comparison reveals that the ISOA exhibits significant enhancements in phase sensitivity and minimum reflectivity compared to the standard SOA, while also requiring fewer iterations to converge. Consequently, the ISOA offers improved precision and efficiency in optimizing multi-layer SPR biosensors.

**Figure 8:**
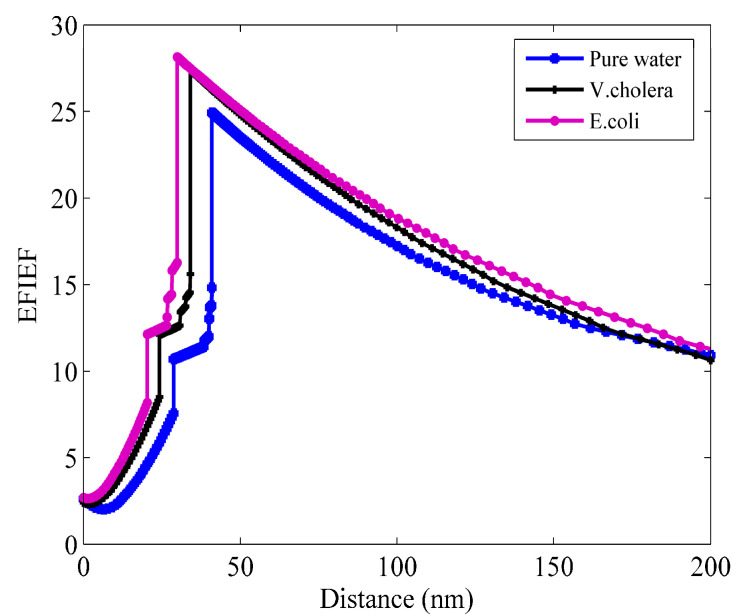
Optimized structure electric field intensity enhancement factor for bacteria detection in water.

In order to further illustrate the feasibility of the methodology proposed in this paper, Figure 8 demonstrates the enhanced electric field intensity factor (EFIEF) to effectively showcase the high-phase-sensitivity properties of the improved sensor structure discussed in this paper. From the figure, it can be seen that after coating the Franckeite and WS_2_ layer on the surface of the conventional SPR sensor, there is great improvement in the electric field intensity, which means the intense excitement of SPs. At the same time, the graph clearly illustrates that the sensing structure with greater sensitivity is likewise associated with a higher electric field intensity.

## 5. Conclusions

This paper utilizes an intelligent optimization technique to create a highly sensitive SPR biosensor. The biosensor incorporates a metal film, a waveguide layer, Franckeite, and WS_2_ to specifically detect bacteria in water. Results show that by controlling the layer thickness of the sensor, while maintaining a minimum reflectance of less than 0.01, the ISOA achieves higher efficiency and accuracy compared to the conventional and SOA methods. The findings indicate that when the waterborne bacteria is *E. coli*, the optimum phase sensitivity is 2.378 × 10^6^ deg/RIU by Ag (20.36 nm)–TiO_2_ (6.08 nm)–Franckeite (monolayer)–WS_2_ (bilayer) after 30 iterations; when the waterborne bacteria is *V. cholera*, the highest phase sensitivity is 1.950 × 10^6^ deg/RIU by Ag (24.31 nm)–TiO_2_ (6.34 nm)–Franckeite (monolayer)–WS_2_ (bilayer) after 26 iterations; when the analyte is pure water, the maximum phase sensitivity is 1.871 × 10^6^ deg/RIU by Ag (28.72 nm)–TiO_2_ (9.59 nm)–Franckeite (monolayer)–WS_2_ (monolayer) after 27 iterations. Hence, the design concept of this article may provide new directions for improving the performance of biosensors applied in environmental detection.

## Figures and Tables

**Table 1:** The 2D materials’ monolayer thickness and RI at λ=633 nm.

Materials	Monolayer (nm)	RI	Reference
Franckeite	1.8	3.58 + 0.39i	[30]
WS_2_	0.8	4.9 + 0.3124i	[33]

**Table 2:** The RI of three types of waking bacteria at λ=633 nm.

Type of Waterborne Bacteria	RI	Reference
Pure water	1.333	[45]
*V. cholera*	1.365	[46]
*E. coli*	1.388	[47]

**Table 3:** The settings of the initialization parameter of the algorithm.

Parameters	Algorithm	
	SOA	ISOA
Population size	100	100
Maximum iterative times	100	100
Maximum degree of membership	0.95	0.95
Minimum degree of membership	0.0111	0.0111
Inertia weight coefficient range	[0.1, 0.9]	/

**Table 4:** The parameters of the optimized Ag–TiO_2_–Franckeite–WS_2_ structure via the SOA.

Waterborne Bacteria	Ag (nm)	TiO_2_ (nm)	Franckeite (N)	WS_2_ (L)	Minimum Reflectivity	Phase Sensitivity (deg/RIU)	Iterations
Pure water	26.75	10.33	1	1	3.373 × 10^−6^	1.841 × 10^6^	79
*V. cholera*	26.30	7.45	1	1	2.307 × 10^−7^	1.909 × 10^6^	70
*E. coli*	18.23	7.54	1	2	9.455 × 10^−6^	2.355 × 10^6^	74

**Table 5:** The parameters of the optimized Ag–TiO_2_–Franckeite–WS_2_ structure via the ISOA.

Waterborne Bacteria	Ag (nm)	TiO_2_ (nm)	Franckeite (N)	WS_2_ (L)	Minimum Reflectivity	Phase Sensitivity (deg/RIU)	Iterations
Pure water	28.72	9.59	1	1	2.058 × 10^−6^	1.871 × 10^6^	27
*V. cholera*	24.31	6.34	1	2	4.957 × 10^−6^	1.950 × 10^6^	26
*E. coli*	20.36	6.08	1	2	1.307 × 10^−6^	2.378 × 10^6^	30

## Data Availability

No new data were created or analyzed in this study. Data sharing is not applicable to this article.

## References

[1] Wu L., Chu H.S., Koh W.S., Li E.P. (2010). Highly sensitive graphene biosensors based on surface plasmon resonance. Opt. Express.

[2] Maharana P.K., Srivastava T., Jha R. (2014). On the performance of highly sensitive and accurate graphene-on-aluminum and silicon-based SPR biosensor for visible and near infrared. Plasmonics.

[3] Bianco M., Sonato A., Girolamo A.D., Pascale M. (2017). An aptamer-based SPR-polarization platform for high sensitive OTA detection. Sens. Actuators B Chem..

[4] Zakaria R., Zainuddin N.A.M., Raya S.A., Alwi S.A.K., Anwar T., Sarlan A., Ahmed K., Amiri I.S. (2020). Sensitivity comparison of refractive index transducer optical fiber based on surface plasmon resonance using Ag, Cu, and bimetallic Ag-Cu Layer. Micromachines.

[5] Hossain B., Paul A.K., Islam M.A., Rahman M.M. (2022). A highly sensitive surface plasmon resonance biosensor using Snse allotrope and heterostructure of Bluep/MoS_2_ for cancerous cell detection. Optik.

[6] Zhou J., Qi Q., Wang C., Qian Y., Liu G., Wang Y., Fu L. (2019). Surface plasmon resonance (SPR) biosensors for food allergen detection in food matrices. Biosens. Bioelectron..

[7] Prado A.R., Diaz C.A.R., Lyra L.G., Oliveira J.P. (2021). Surface plasmon resonance-based optical fiber sensors for H_2_S in situ detection. Plasmonics.

[8] Guner H., Ozgur E., Kokturk G., Celik M., Esen E., Topal A.E., Ayas S., Uludag Y., Elbuken C., Dana A. (2017). A smart phone-based surface plasmon resonance imaging (SPRi) platform for on-site biodetection. Sens. Actuator B Chem..

[9] Rossi S., Gazzola E., Capaldo P., Borile G., Romanato F. (2018). Grating-Coupled Surface Plasmon Resonance (GC-SPR) Optimization for Phase-Interrogation Biosensing in a Microfluidic Chamber. Sensors.

[10] Kushwaha A.S., Kumar A., Kumar R., Srivastava S.K. (2018). A study of surface plasmon resonance (SPR) based biosensor with improved sensitivity. Photonic. Nanostruct..

[11] Wu L., Jia Y., Jiang L., Guo J., Dai X., Xiang Y., Fan D. (2017). Sensitivity improved SPR biosensor based on the MoS_2_/graphene–aluminum hybrid structure. J. Light. Technol..

[12] Mudgal N., Saharia A., Choure K.K., Agarwal A., Singh G. (2020). Sensitivity enhancement with anti-reflection coating of siliconnitride (Si_3_N_4_) layer in silver-based surface plasmon resonance (SPR) sensor for sensing of DNA hybridization. Appl. Phys. A Mater. Sci. Process..

[13] Yue C., Ding Y.Q., Tao L., Zhou S., Guo Y.C. (2023). Differential evolution particle swarm optimization for phase sensitivity enhancement of SPR gas sensor based on MXene and BlueP/TMDCs hybrid structure. Sensors.

[14] Aref S.H. (2018). SPR phase sensitivity enhancement in common-path polarization heterodyne interferometer by polarization tuning. Optik.

[15] Wang R., Du L.P., Zhang C.L. (2013). Plasmonic petal-shaped beam for microscopic phase-sensitive SPR biosensor with ultrahigh sensitivity. Opt. Lett..

[16] Yue C., Qin Z.R., Lang Y.P., Liu Q.G. (2019). Determination of thin metal film’s thickness and optical constants based on SPR phase detection by simulated annealing particle swarm optimization. Opt. Commun..

[17] Liu C., Liu Q., Qin Z. (2017). Determination of the bimetallic layers’ film thicknesses by phase detection of SPR prism coupler. Plasmonics.

[18] Li Y., Zhang Y., Chen Q. (2020). Surface plasmon resonance effect, nonlinearity and faraday rotation properties of magneto optical glass: Influence of diamagnetic Ag@ZrO_2_ nanoparticles. J. Non-Cryst. Solids.

[19] Hoa X.D., Kirk A.G., Tabrizian M. (2007). Towards integrated and sensitive surface plasmon resonance biosensors: A review of recent progress. Biosens. Bioelectron..

[20] Vincenzo A., Roberto P., Marco F. (2017). Surface plasmon resonance in gold nanoparticles: A review. J. Phys. Condens. Matter..

[21] Verma R., Gupta B.D., Jha R. (2011). Sensitivity enhancement of a surface plasmon resonance-based biomolecules sensor using graphene and silicon layers. Sens. Actuators B Chem..

[22] Shukla S., Sharma N.K., Sajal V. (2015). Sensitivity enhancement of a surface plasmon resonance based fiber optic sensor using Zno thin film: A theoretical study. Sens. Actuators B Chem..

[23] Lin Z., Shu Y., Chen W., Zhao Y., Li J. (2022). High sensitivity PtSe2 surface plasmon resonance biosensor based on metal-Si-metal waveguide structure. Biosensors.

[24] Wang Z., Yu M., Li K., Mao H., Liu K., Li H. (2022). Tunable fano resonance-enhanced surface plasmon biosensor based on Mxene/MoS_2_ heterostructure. Opt. Mater..

[25] Deng Y., Li M., Cao W., Wang M., Hao H., Xia W. (2021). Fiber optic coupled surface plasmon resonance sensor based Ag-TiO_2_ films for hydrogen detection. Opt. Fiber Technol..

[26] Maurya J.B., Prajapati Y.K. (2017). Influence of dielectric coating on performance of surface plasmon resonance sensor. Plasmonics.

[27] Nesterenko D.V., Sekkat Z. (2013). Resolution estimation of the Au, Ag, Cu, and Al single- and double-layer surface plasmon sensors in the ultraviolet, visible, and infrared regions. Plasmonics.

[28] Gant P., Ghasemi F., Maeso D., Munuera C. (2017). Optical contrast and refractive index of natural van der waals heterostructure nanosheets of franckeite. Beilstein J. Nanotechnol..

[29] Karki B., Sharma S., Singh Y., Pal A. (2022). Sensitivity enhancement of surface plasmon resonance biosensor with 2-D franckeite nanosheets. Plasmonics.

[30] Yesudasu V., Pradhan H.S., Pandya R.J., Thiyaneswaran B. (2023). Numerical investigation of Ag-Franckeite-Barium Titanium-BP based highly performed surface plasmon resonance sensor for virus SARS-CoV-2 detection. Plasmonics.

[31] Srivastava A., Prajapati Y.K. (2020). Effect of sulfosalt and polymers on performance parameter of SPR biosensor. Opt. Quantum Electron..

[32] Gan S., Zhao Y., Dai X., Xiang Y. (2019). Sensitivity enhancement of surface plasmon resonance sensors with 2d franckeite nanosheets. Res. Phys..

[33] Zakaria R., Zainuddin N.A.A.M., Leong T.C., Rosli R., Rusdi M.F., Harun S.W., Sadegh Amiri I. (2019). Investigation of surface plasmon resonance (SPR) in MoS_2_-and WS_2_-protected titanium side-polished optical fiber as a humidity sensor. Micromachines.

[34] Rahman M.S., Anower M.S., Abdulrazak L.F. (2019). Utilization of a phosphorene-graphene/TMDC heterostructure in a surface plasmon resonance-based fiber optic biosensor. Photonic. Nanostruct..

[35] Wu L.M., Guo J., Wang Q.K., Lu S.B., Dai X.Y., Xiang Y.J. (2017). Sensitivity enhancement by using few-layer black phosphorus-graphene/TMDCs heterostructure in surface plasmon resonance biochemical sensor. Sens. Actuat B Chem..

[36] Zeng S., Hu S., Xia J., Anderson T., Dinh X.Q., Meng X.M. (2015). Graphene-MoS_2_ hybrid nanostructures enhanced surface plasmon resonance biosensors. Sens. Actuators B.

[37] Han L., He X.J., Huang T.Y. (2019). Comprehensive study of SPR biosensor performance based on metal-ITO-graphene/TMDCs hybrid multilayer. Plasmonic.

[38] Xia G., Zhou C., Jin S., Huang C., Xing J., Liu Z. (2019). Sensitivity enhancement of two-dimensional materials based on genetic optimization in surface plasmon resonance. Sensors.

[39] Amoosoltani N., Zarifkar A., Farmani A. (2019). Particle swarm optimization and finite-difference time-domain (pso/fdtd) algorithms for a surface plasmon resonance-based gas sensor. J. Comput. Electron..

[40] Sun Y., Cai H., Wang X., Zhan S. (2018). Optimization methodology for structural multiparameter surface plasmon resonance sensors in different modulation modes based on particle swarm optimization. Opt. Commun..

[41] Lin Z., Chen S., Lin C. (2020). Sensitivity improvement of a surface plasmon resonance sensor based on two-dimensional materials hybrid structure in visible region: A theoretical study. Sensors.

[42] Dai C.H., Chen W. (2006). Seeker optimization algorithm. International Conference on Computational and Information. Science.

[43] Su H., Yang J. (2013). Capacitors optimization placement in distribution systems based on improved seeker optimization algorithm. Sens. Transducers.

[44] Duan S.M., Luo H., Liu H. (2022). A complex-valued encoding multichain seeker optimization algorithm for engineering problems. Sci. Program..

[45] Sylvester-Hvid K.O., Mikkelsen K.V., Ratner M.A. (2011). The iterative self-consistent reaction-field method: The refractive index of pure water. Int. J. Quantum Chem..

[46] Liu P.Y., Chin L.K., Ser W., Ayi T.C., Yap P.H., Bourouina T., Leprince-Wang Y. Real-time measurement of single bacterium’s refractive index using optofluidic immersion refractometry. Proceedings of the 28th European Conference on Solid-StateTransducers (Eurosensors 2014).

[47] JWaswa W., Debroy C., Irudayaraj J. (2006). Rapid detection of Salmonella enteritidis and Escherichia coli using surface plasmon resonance biosensor. J. Food Process Eng..

[48] Singh M.K., Pal S., Verma A., Prajapati Y.K., Saini J.P. (2020). Highly sensitive antimonene-coated black phosphorous-based surface plasmon-resonance biosensor for DNA hybridization: Design and numerical analysis. J. Nanophotonics.

[49] Kumar D., Samantaray S.R. (2016). Implementation of multi-objective seeker-optimization-algorithm for optimal planning of primary distribution systems including dstatcom. Int. J. Electr. Power Energy Syst..

[50] Tuba M., Bacanin N. (2014). Improved seeker optimization algorithm hybridized with firefly algorithm for constrained optimization problems. Neurocomputing.

